# The Effect of Positive Interactions on Temporal Turnover of Community Composition along an Environmental Gradient

**DOI:** 10.1371/journal.pone.0078698

**Published:** 2013-11-12

**Authors:** Youshi Wang, Zhiyong Yang, Shurong Zhou, Janne Soininen, Dexiecuo Ai, Yali Li, Chengjin Chu

**Affiliations:** 1 Ministry of Education Key Laboratory of Western China’s Environmental Systems, Research School of Arid Environment and Climate Change, Lanzhou University, Lanzhou, China; 2 State Key Laboratory of Grassland and Agro-Ecosystems, School of Life Sciences, Lanzhou University, Lanzhou, China; 3 Ministry of Education Key Laboratory for Biodiversity Science and Ecological Engineering, School of life Sciences, Fudan University, Shanghai, China; 4 Department of Geosciences and Geography, University of Helsinki, Helsinki, Finland; 5 Xikehe Sire Breeding Farm of Euler Sheep of Maqu County in the Gansu Province, Maqu, China; University of Sydney, Australia

## Abstract

It has been demonstrated that the interplay between negative and positive interactions simultaneously shapes community structure and composition. However, few studies have attempted to examine the effect of facilitation on compositional changes in communities through time. Additionally, due to the difficulties in collecting the long-term data, it would be useful to indicate the rate of temporal turnover using a readily obtainable metric. Using an individual-based model incorporating plant strategies, we examined the role of facilitation on the temporal turnover of communities located at different positions along an environmental gradient for three model scenarios: CM without facilitation; CFM-U, a unimodal relationship between facilitation and environmental severity; and CFM-L, a positively linear relationship between facilitation and environmental severity. Our results demonstrated that facilitation could increase, decrease or have no remarkable effect on temporal turnover. The specific outcome depended on the location of the focal community across the environmental gradient and the model employed. Compared with CM, the inclusion of positive interactions (i.e. CFM-U and CFM-L), at intermediate environmental stress levels (such as *S = *0.7 and 0.8) resulted in lower Bray-Curtis similarity values; at other severity levels, facilitation slowed down (such as *S = *0.3 and 0.4 at low to medium stress levels, and *S = *0.9 at high stress levels) or had only a subtle effect (such as at *S = *0.1) on temporal turnover. We also found that the coefficient of variation (CV) in species abundances and the rate of temporal variability showed a significant quadratic relationship. Our theoretical analysis contributes to the understanding of factors driving temporal turnover in biotic communities, and presents a potential metric (i.e. CV in species abundances) assessing the consequences of ongoing environmental change on community structure.

## Introduction

Compositional turnover of communities has attracted more attention in recent years. Such studies help to unravel the causes and consequences of biodiversity in nature, and the patterns of compositional changes provide valuable information to many ecological and evolutionary questions, as well as to conservation planning [1]–[8]. It has been suggested that addressing biotic community turnover can no longer be approached soley from a spatial aspect, but also be understood from a temporal aspect. This is because projected environmental change in the future is expected to cause large shifts both in species spatial distributions, and in temporal assemblage patterns [9], [10].

Though the spatial dataset of community composition/turnover has been well collected and documented, the scarcity of temporal data has hindered the comprehensive exploration of potential factors contributing to the compositional variation through time, especially for terrestrial communities [5]. It has been proposed that like the spatial variation, temporal variability is also likely to be driven by multiple biotic and abiotic factors [9], [11]–[13]. For example, Korhonen et al. [9] demonstrated that sampling duration, body size, ecosystem size and type, and latitude correlated with the observed degree of temporal variation in aquatic assemblages. Adler et al. [11] reported that the general pattern of the species-time-area relationship was derived from demographic processes and ecological interactions which played out on a template of environmental variation. Wang et al. [13] argued that at the level of entire communities, facilitation could make species more prone to extinction on average due to the smaller mean population size (i.e. the total number of individuals divided by species richness) [14]–[18] and thus increase the temporal turnover in communities. Compared with other biotic and abiotic factors, however, the role of facilitation has been overlooked in the studies of compositional changes in time [13].

Studies over the last 20 years have shown that positive interactions are common in plant communities in physically harsh conditions, through ameliorating locally stressful environments by increasing temperature in alpine regions [13], [19]–[21] and increasing soil water content in arid and semi-arid areas [22], [23]. Though a growing amount of work has been conducted to test the effect of facilitation on population dynamics [24]–[30], community structure [21], [31], [32], and ecosystem functioning [33]–[35], to our knowledge, few studies have investigated the potential impact of positive interactions on the temporal turnover of community composition [13]. As the occurrence and magnitude of positive interactions are closely associated with the environmental conditions and species-specific life-history characteristics, exploring the potential role of facilitation on temporal turnover could shed light on the consequences of ongoing global change [36]–[38].

Comparing to rigorously controlled experiments, simulation modeling renders us a more effective and practical tool for assessing the impact of biotic interactions on community properties such as turnover through time. Using birth-death tradeoff models, our previous work indicated that facilitation increased compositional turnover through time: communities with facilitation were more dissimilar than neutral communities after the same simulation time [13]. However, a snapshot at one point on an environmental continuum was not sufficient to make robust conclusions on the general patterns [39]. Additionally, it is difficult to include other species properties into birth-death tradeoff models. For instance, it has been demonstrated that the strength and sign of plant-plant interactions depend strongly on the life-history strategies of the interacting species, i.e. whether a species is competitive or stress-tolerant [13], [19], [40].

Additionally, due to the difficulties in collecting the long-term data, it would be useful to indicate the rate of temporal turnover using a readily obtainable metric. Though Wang et al. [13] proposed that the mean population size might provide such a metric, the mean population size *per se* could not tease apart the community with numerous species having very low abundances from the community with many species with medium abundances. The coefficient of variation (CV) in species abundances defined as the ratio of the standard deviation to the mean of species abundances in a community could be a potential candidate for such a metric.

In this paper, we used an individual-based spatially explicit simulation model to study theoretically the pattern of temporal turnover in communities along an environmental gradient, taking into account biotic interactions and species’ life-history strategies. In order to simulate the simultaneous negative and positive interactions in the real communities, we constructed a model includes both competition and facilitation (hereafter CFM) to explore the temporal turnover along the environmental gradient. To investigate the potential effect of positive interactions on compositional changes through time, we compared the results from CFM with ones from a model without facilitation (hereafter CM). Specifically, we asked the following three questions: 1) How does the temporal turnover in communities change across the environmental gradient? 2) What are the effects of facilitation on temporal turnover, increasing, decreasing or having no impact at all? And 3) Does CV in species abundances provide a useful metric for indicating the rate of temporal turnover?

## Results

**Figure 1:**
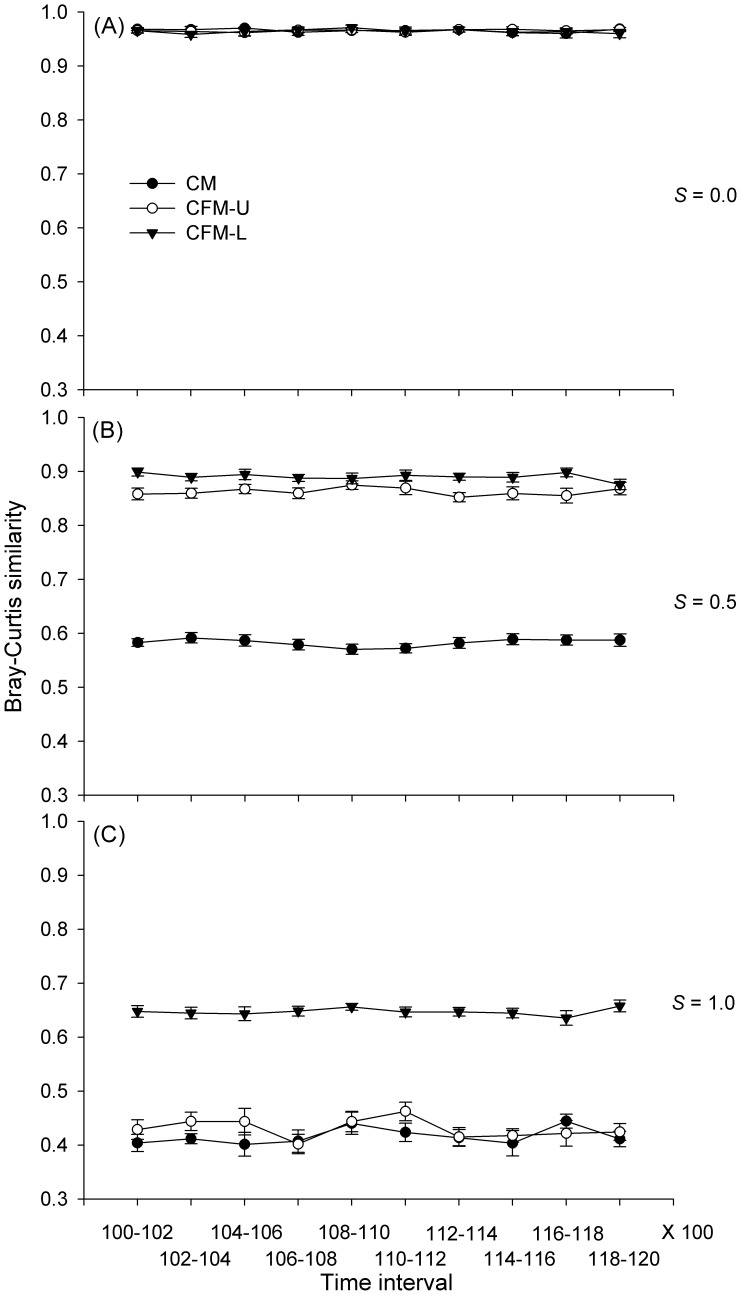
Changes of community composition with the same simulation time (here the time interval is 200 steps) for three environmental levels: low (*S* = 0.0; A), medium (*S* = 0.5; B) and high (*S* = 1.0; C). For each environmental level, three models were presented: CM, CFM-U, and CFM-L. The parameter values used in models are *r_max_*_ = _1, *r_min_*_ = _0.2, *f* = 0.5, *S_m_*_ = _0.8, *I* = 30, *d* = 0.2, *r_s_* = −0.1 and *c* = 1. Each data point represents the mean±SE.

For each model scenario, under the benign condition (*S = *0.0), the Bray-Curtis similarity value between communities after 200 simulation steps was about 0.96 (Fig. 1). At the intermediate environmental stress (*S = *0.5), CM communities had smaller similarity values than CFM-U and CFM-L communities. In extremely stressful conditions (*S = *1.0), CFM-L communities had higher similarity values than CM and CFM-U communities, indicating that facilitation slowed down the community turnover through time in highly harsh conditions.

**Figure 2:**
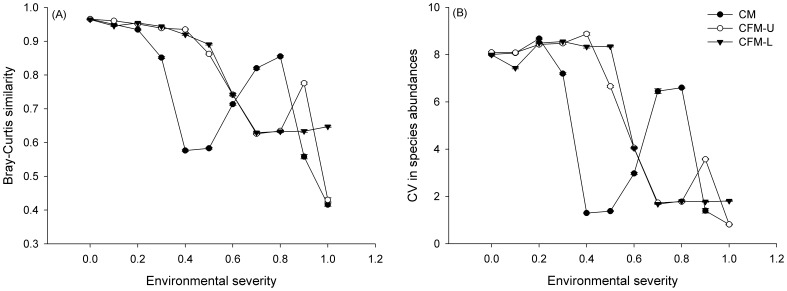
The Bray-Curtis similarity (A) and the coefficient of variation in species abundances (B) along the environmental gradient for communities with (CFM-U, and CFM-L) and without (CM) facilitation. The parameter values are the same as in Fig. 1. Each data point represents the mean±SE.

After the same simulation period (200 steps), Bray-Curtis similarity values for CFM-U and CFM-L models were similar when stress levels were less than 0.9 (Fig. 2). Compared with CM, the inclusion of positive interactions (i.e. models CFM-U and CFM-L) resulted in lower Bray-Curtis similarity values at intermediate environmental stress levels (Fig. 2A; for example, at *S = *0.7 and 0.8). For other severity levels, facilitation slowed down (for example *S = *0.3 and 0.4 at low to medium stress levels, and *S = *0.9 at high stress levels) or had only a subtle effect (for example, *S = *0.1) on temporal turnover. At two extreme ends of the gradient (*S = *0.0, and 1.0), facilitation played no role on species turnover between CM and CFM-U communities.

**Figure 3:**
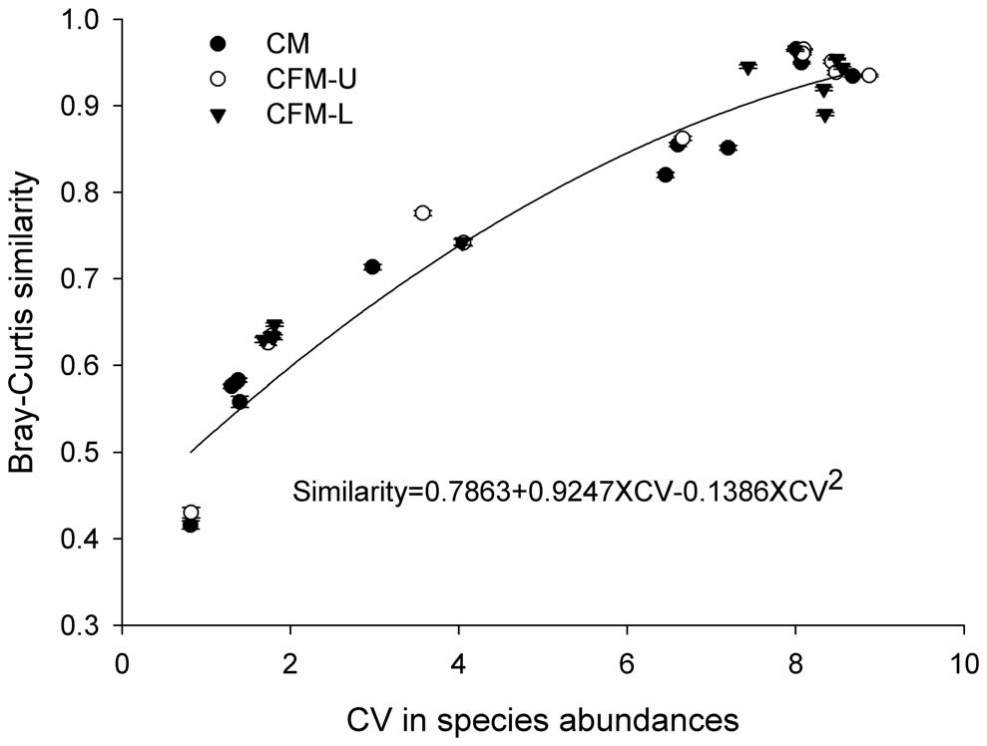
The relationship between the coefficient of variation in species abundances and the Bray-Curtis similarity index for communities with (CFM-U, and CFM-L) and without (CM) facilitation. The parameter values are the same as in Fig. 1. Each data point represents the mean±SE.

The CV in species abundances showed similar patterns with compositional changes for communities along the environmental gradient (Fig. 2B). A quadratic regression model well described the relationship between CV in species abundance and Bray-Curtis similarity. The model scenarios did not affect the correlations between CV in species abundance and Bray-Curtis similarity (Fig. 3), with the R^2^ = 0.9498.

## Discussion

Along the environmental gradient, CM communities without facilitation showed the pattern of temporal turnover with three distinct sections: Bray-Curtis similarity values first decreased, then increased and lastly decreased again (Fig. 2A). CFM-U communities displayed a similar three-sectioned pattern (Fig. 2A). In contrast, CFM-L communities exhibited a pattern with two sections: Bray-Curtis similarity values first decreased from benign (*S = *0.0) to harsh (*S = *0.7) conditions, then slightly increased through highly stressful environments (*S = *0.7 to 1.0). These results indicated that a simple linear relationship between temporal turnover and environmental conditions may not exist. It has been reported that the degree of compositional turnover through time showed large-scale environmental and geographical variation, such as along latitude [9], [41]. For example, it has been suggested that communities in tropics with benign conditions should have higher temporal stability [41]. Our results overall are consistant with these reported findings, however, more studies are needed to validate the found nonlinear pattern in temporal turnover along environmental severity in the field. Fruitful avenues for such studies could be to focus on communities sharing a common species pool along an elevational gradient or along a single environmental variable such as salinity or temperature.

In our previous work based on birth-death tradeoff models, we elucidated that positive interactions in a saturated community could fasten the compositional turnover [13]. Obviously, this result represented a special case with the strict assumption of community saturation and may not reflect the general pattern in turnover. The present study, however, displayed a full range of environmental conditions without the limitation of saturation. Our results indicated that the effect of facilitation on temporal turnover depended on the location of the focal community across the environmental gradient and the model involved (Fig. 2A). From benign (*S = *0.0) to intermediately stressful (*S = *0.6) conditions, facilitation slowed down the compositional changes through time: both CFM-U and CFM-L communities had higher similarity values than CM communities. Conversely, from the severity level of 0.7 to 0.8, facilitation fastened the temporal turnover: CM communities had higher similarity values than communities with facilitation. At *S = *0.9, CFM-U communities had the slowest turnover rate, and CM communities had the fastest turnover rate. Under the extremely stressful conditions (*S = *1.0), no difference on compositional changes between CFM-U and CM communities was found. However, the strongly linear increase of facilitation with stress levels in CFM-L communities resulted in a slower compositional change through time (Fig. 2A). The differences on compositional turnover between CFM-U and CFM-L communities mainly stemmed from the different model settings: the former assumed a unimodal relationship between facilitation and severity, and the latter assumed a positively linear relationship between two variables. Based on the observed temporal turnover of communities along an environmental gradient, researchers could identify the underlying relationship between facilitation and severity levels. For instance, if communities follow a two-sectioned pattern of temporal turnover, according to our simulations, a positively linear relationship between facilitation and severity might be prevalent, which presents another potential way to test the Stress-Gradient Hypothesis [42]–[44].

The pattern of CV in species abundances displayed a similar trend with the change of similarities (Fig. 2), which implied that CV in species abundances could indicate the compositional turnover through time. Our analysis further demonstrated that there was a quadratic relationship between CV in species abundances and temporal turnover (Fig. 3): Communities with larger CV in species abundances had slower temporal turnover. This quadratic curve well explained the pattern of temporal turnover of CFM along the environmental gradient, and the difference between CFM and CM.

Another interesting point we should emphasize is that such quadratic curve is not dependent on the models involved, i.e. CFM and CM showed nearly similar patterns. This implies that the CV in species abundances could be a useful metric indicating the rate of temporal turnover for real communities: rapid temporal turnover could occur in communities with small CV in species abundances. In other words, communities with species with relatively even abundances could have faster temporal turnover than communities dominated by one or several species with others having low abundances. Though it is difficult to determine the specific mechanisms underpinning temporal turnover from CV in species abundances alone, we can make a preliminary judgment about the rate of compositional change in time for given communities, and corresponding assessment on community structure and functional stability of the ecosystems. For instance, fast temporal turnover in community composition could manifest the instability of ecosystem functioning.

To conclude, this paper presents our first attempt to theoretically explore the patterns in temporal turnover along a full environmental gradient, and such an attempt can contribute uniting the studies about spatial and temporal aspects of biodiversity [11], [12], [45]. We demonstrated that the effect of positive interactions among plants on compositional changes through time depends on the relationship between the environmental gradient and facilitation, and the position of the community involved across the environmental gradient. We advocate that more observational/experimental studies should be conducted to evaluate the results emerged here, especially focusing on terrestrial communities.

## Materials and Methods

Our model is an extention of Xiao et al.’s [32] competitive/stress-tolerance tradeoff model. In the model, the environmental gradient is indicated by the variable *S*, which ranges from 0 (i.e. most benign environment) to 1 (i.e. most severe environment) with the interval 0.1. Totally, we have 11 environmental levels. Competitive ability of species *i* is characterized by the variable *p_i_* (0≤*p_i_*≤1), which was randomly drawn from a uniform distribution ([0, 1]) [32]. The larger the *p_i_* is, the higher is the probability (*p_i_−p_j_*) that species *i* invades a neighboring cell occupied by another species *j*. We also assume that any species can invade the empty cells. To account for the tradeoff, we assume that the reproduction rate (dedicated by the variable *r*) of competitive species declines more rapidly with increasing environmental stress than that of stress-tolerant species. Thus, the reproduction rate of species *i* when considering only the negative interactions (the CM model) is:
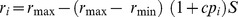
(1)where *r_max_* and *r_min_* are the maximum and minimum values of reproduction, corresponding to the benign and harsh ends of the environmental gradient, respectively. Though it is possible to set species-specific *r_max_* and *r_min_*, we have no sufficient prior information from literature to determine the relationships between *r_max_* and *r_min_* and corresponding competitive ability of species. We thus assume that all species share the identical community-level *r_max_* and *r_min_*. Here, *c* is a constant larger than zero, indicating that the reproduction rate is linearly decreasing with the species’ competitive ability. Additionally, we set a threshold *r_s_*, below which individuals will die [32].

Different performance currencies have been used to assess the effect of facilitation [20], [44], [46], such as vital demographic rates, and population density. In the present paper, we assume that the facilitation can increase species’ reproduction rate [20], [32]. With regard to the relationship between facilitation and environmental gradients, various scenarios have emerged from the theoretical and experimental studies. Firstly, it has been demonstrated that the balance between competition and facilitation shifts from negative to positive with the increasing magnitude of environmental severity [42], [43], [47]. This has been synthesized in the “Stress-Gradient Hypothesis” (SGH) with a linear and positive relationship between facilitation and environmental gradients (the CFM-L model). Meanwhile, Michalet et al. [33] theoretically proposed that the role of facilitation could diminish in the most severe conditions from a threshold of environmental severity level *S_m_* (*S_m_ = *0.8 in the present paper; the concrete value of *S_m_* does not qualitatively influence the results, see Fig. S1 and Fig. S2 for the case of *S_m_ = *0.9) to reach zero when *S = *1 (i.e. under the most severe conditions), where a unimodal relationship between facilitation and environmental gradients was found (the CFM-U model) [23]. Additionally, other studies which mainly focused on the nurse cushion plants (i.e. the cushion plants were treated as benefactors) demonstrated that net facilitation remained constant or even decreased along stress gradients [48]–[52]. Differing from heterospecific facilitative interactions explored in Xiao et al. [32] which mainly simulated nurse effects in nature, here we consider both intra- and interspecific facilitation. For example, in communities such as alpine meadows, the aggregation of plants regardless of species identity could increase the local temperature and prevent the physical damage from strong wind [20]. Thus in the present paper we will not take account of the scenario with constant or decreasing relationships of facilitation with stress gradients. Finally, we conducted simulations for three types of models: CM (i.e. without facilitation), CFM-U (the unimodal relationship between facilitation and the environmental gradient), and CFM-L (the linear relationship between facilitation and the environmental gradient).

For the CFM-U scenario, the reproduction rate including both competition and facilitation is
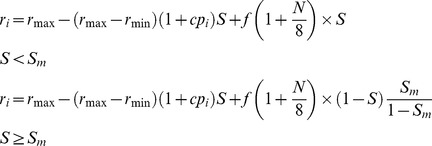
(2)


Correspondingly, we have the following formula for the reproduction rate for the CFM-L scenario
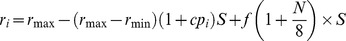
(3)where *f* is the facilitative coefficient (we set *f = *0.5 here; the specific value of *f* does not qualitatively influence the results, see Fig. S3 and Fig. S4 for the case of *f* = 0.3), indicating the intensity of positive effect received by a focal individual from the presence of neighbors; *N* is the number of neighbors, which are located in the closest eight neighboring cells (i.e. Moore neighborhood), indicating the localized property of interactions for plants [53]. When there were no individuals occupying the neighboring cells of a focal plant, i.e. *N = *0, the parameter *f* was set to 0, and then Eqn 2 and 3 were equal to Eqn 1. In the original model [32], for a target individual, it assumed facilitation was dependent on the presence/absence of neighbors regardless of how many neighbors were surrounding. To make the model more reasonable and consistent with the experiments [19], here we propose that the effect of facilitation is additive, i.e. linear with the number of neighbors [13], [24], [26].

Simulations were implemented on a landscape with the size of 100×100 grids. At a given environmental level (i.e. a specific *S* value), all individuals on this landscape shared the identical *S* value. The initial community was saturated and occupied by the same number of species as in regional species pool (*R = *200; using *R = *100, we obtained qualitatively similar results with the ones presented here, see Fig. S5 and Fig. S6), with the number of individuals for each species followed a negative exponential distribution [54]. Then the model was sequentially run through the following modules: deaths, immigration, and reproduction.

*Deaths*. All individuals within the community experience the identical probability of death *d*, which simulates the effect of environmental stochasticity and disturbance [32].

*Immigration*. To make our model more realistic and reasonable, a fixed number of species (*I = *30; results are qualitatively similar with *I = *20, see Fig. S7 and Fig. S8) are randomly drawn from the species pool, and randomly dispersed to the local community [32]. When the species arrives at a cell already occupied by other species, relative competitive ability between two species determines the final outcome. In the case of two species *i* (immigrant species) and *j* (resident species), for example, if *p_i_−p_j_* is bigger than a random number from [0, 1], species *i* will replace species *j*.

*Reproduction*. For a given focal individual, with and without considering facilitation, the reproduction rate *r_i_* is calculated using the above corresponding equation. Due to the fact that *r_i_* is not always an integer, when *r_i_* >0, the number of offsprings identical to their parent is estimated as following: if the decimal of *r_i_* is larger than a random number drawn from [0, 1], the offspring number will be the integer of *r_i_* plus 1. Each propagule is then dispersed to one of the cells on the landscape according to a dispersal kernel *K*_2Dt_ taking the form [55].
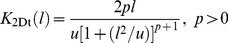
(4)where *u* and *p* are parameters determining the shape of the function. This kernel combines Gaussian dispersal at short distances with a power-law tail of long-distance dispersal [55]. In the present simulations, we set *u = *10 and *p = *1. If the designated cell for dispersal has been occupied by other species, competition will determine the success probability of invasion following the similar rule of immigration described above.

All simulations were run for 10 000 time steps in order to allow the community to reach a steady state. For each model scenario, community composition including species identity and abundance were recorded with the interval of 200 steps for each environmental level after the 10 000 startup steps (10 000, 10 200, 10 400, and so on). For each parameter setting, total 10 intervals consisiting of 11 communities were recorded until 12 000 steps. To describe the compositional changes of communities, Bray-Curtis similarity index accounting for both the presence/absence and the abundance of each species was chosen [5], [13], [56]–[59]. To facilitate the exploration of potential metrics indicating the rate of temporal turnover, rather than comparing the compositional changes of communities with the original community along simulation time, we sequentially calculated the Bray-Curtis similarity for communities at two ends of each interval, with the previous community as the reference (i.e. the pairwise comparison). For instance, for the CM model, at *S = *0.0, we calculated similarity index between the community at 10 000 and the community at 10 200, the similarity index between the community at 10 200 and the community at 10 400, and so on. Ten replicates were performed for each parameter combination. Finally, we conducted a total of 330 simulations: 10 replicates×11 environmental levels (from 0.0 to 1.0 with the interval 0.1)×3 types of models (CM, CFM-U, and CFM-L). For each environmental level, the Bray-Curtis similarity value was obtained by averaging individual values across 10 intervals and across 10 replicates.

For the community recorded on each time point (i.e. 10000, 10200, 10400, and so on), we calculated the coefficient of variation (CV) in abundances among member species. We used the CV in species abundances for the former community of a time interval to predict the temporal turnover of this community during this time interval. For each environmental level, the CV value was obtained by averaging individual values across 10 communities and across 10 replicates. To test the potential relationship between CV values and Bray-Curtis similarity values along the environmental gradient, based on the simulated patterns, we fit the data using a linear regression model and a quadratic regression model [60]. The measure of the Akaike Information Criterion (AIC) showed that the quadratic regression model had the smaller AIC value than the one from the linear regression model. We also explored the relationship between mean population size and Bray-Curtis similarity (Fig. S9).

Simulations were implemented in NetLogo software [61], and a ‘wraparound’ approach (i.e. periodic boundary conditions) was used to avoid edge effects [53]. The Bray-Curtis similarity index was calculated in the package *fossil* [62] on the R platform [60].

## Supporting Information

Figure S1 The Bray-Curtis similarity (A) and the coefficient of variation in species abundances (B) along the environmental gradient for communities with (CFM-U, and CFM-L) and without (CM) facilitation. The threshold of environmental severity level *S_m_* = 0.9, and other parameter values are the same as in Fig. 1. Each data point represents the mean±SE.
(TIF)

Figure S2 The relationship between the coefficient of variation in species abundances and the Bray-Curtis similarity index for communities with (CFM-U, and CFM-L) and without (CM) facilitation. The threshold of environmental severity level *S_m_* = 0.9, and other parameter values are the same as in Fig. 1. Each data point represents the mean±SE. The R^2^ of the quadratic regression is 0.9395.
(TIF)

Figure S3 The Bray-Curtis similarity (A) and the coefficient of variation in species abundances (B) along the environmental gradient for communities with (CFM-U, and CFM-L) and without (CM) facilitation. The facilitative coefficient *f* = 0.3, and other parameter values are the same as in Fig. 1. Each data point represents the mean±SE.
(TIF)

Figure S4 The relationship between the coefficient of variation in species abundances and the Bray-Curtis similarity index for communities with (CFM-U, and CFM-L) and without (CM) facilitation. The facilitative coefficient *f* = 0.3, and other parameter values are the same as in Fig. 1. Each data point represents the mean±SE. The R^2^ of the quadratic regression is 0.9230.
(TIF)

Figure S5 The Bray-Curtis similarity (A) and the coefficient of variation in species abundances (B) along the environmental gradient for communities with (CFM-U, and CFM-L) and without (CM) facilitation. The regional species pool *R* = 100, and other parameter values are the same as in Fig. 1. Each data point represents the mean±SE.
(TIF)

Figure S6 The relationship between the coefficient of variation in species abundances and the Bray-Curtis similarity index for communities with (CFM-U, and CFM-L) and without (CM) facilitation. The regional species pool *R* = 100, and other parameter values are the same as in Fig. 1. Each data point represents the mean±SE. The R^2^ of the quadratic regression is 0.9274.
(TIF)

Figure S7 The Bray-Curtis similarity (A) and the coefficient of variation in species abundances (B) along the environmental gradient for communities with (CFM-U, and CFM-L) and without (CM) facilitation. The immigration rate *I* = 20, and other parameter values are the same as in Fig. 1. Each data point represents the mean±SE.
(TIF)

Figure S8 The relationship between the coefficient of variation in species abundances and the Bray-Curtis similarity index for communities with (CFM-U, and CFM-L) and without (CM) facilitation. The immigration rate *I* = 20, and other parameter values are the same as in Fig. 1. Each data point represents the mean±SE. The R^2^ of the quadratic regression is 0.9256.
(TIF)

Figure S9 The relationship between the mean population size (MPS) and the Bray-Curtis similarity index for communities with (CFM-U, and CFM-L) and without (CM) facilitation. The parameter values are the same as in Fig. 1. Each data point represents the mean±SE. The R^2^ of the quadratic regression is 0.8585.
(TIF)
