# Supplementary figures and images for: The Effect of Positive Interactions on Temporal Turnover of Community Composition along an Environmental Gradient
Source: PLoS One. 2013 Nov 12;8(11):e78698. doi: 10.1371/journal.pone.0078698 (PMC3827060; doi:10.1371/journal.pone.0078698)

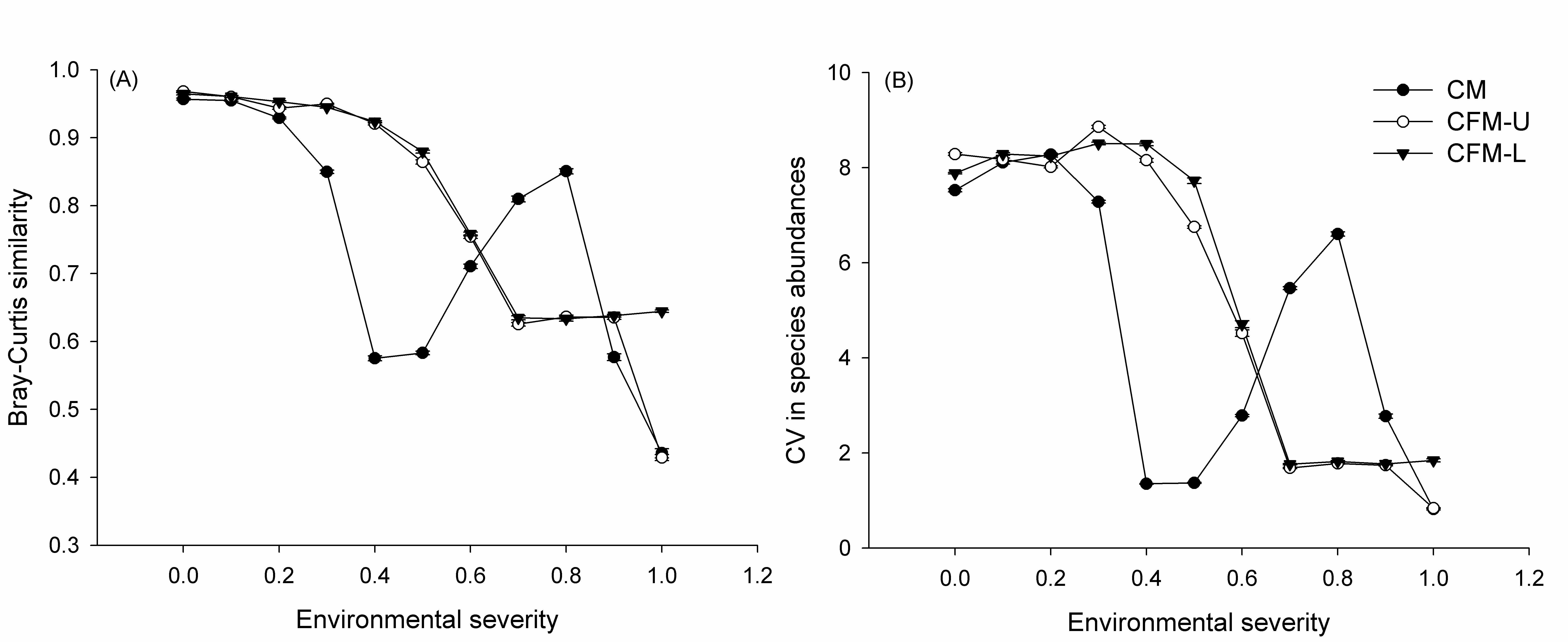

Supplement: Figure S1 — The Bray-Curtis similarity (A) and the coefficient of variation in species abundances (B) along the environmental gradient for communities with (CFM-U, and CFM-L) and without (CM) facilitation. The threshold of environmental severity level Sm = 0.9, and other parameter values are the same as in Fig. 1. Each data point represents the mean±SE. (TIF) [file pone.0078698.s001.tif]

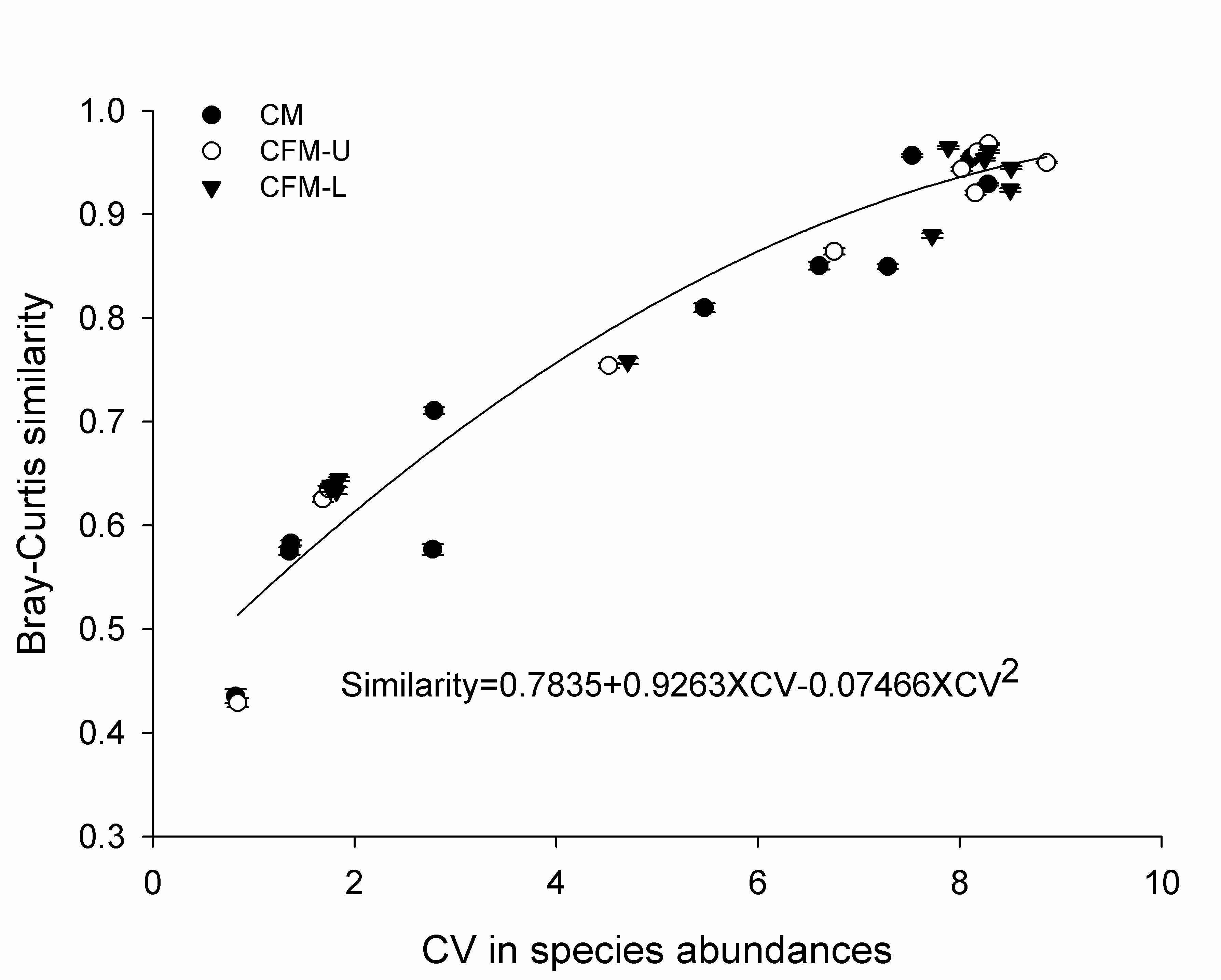

Supplement: Figure S2 — The relationship between the coefficient of variation in species abundances and the Bray-Curtis similarity index for communities with (CFM-U, and CFM-L) and without (CM) facilitation. The threshold of environmental severity level Sm = 0.9, and other parameter values are the same as in Fig. 1. Each data point represents the mean±SE. The R2 of the quadratic regression is 0.9395. (TIF) [file pone.0078698.s002.tif]

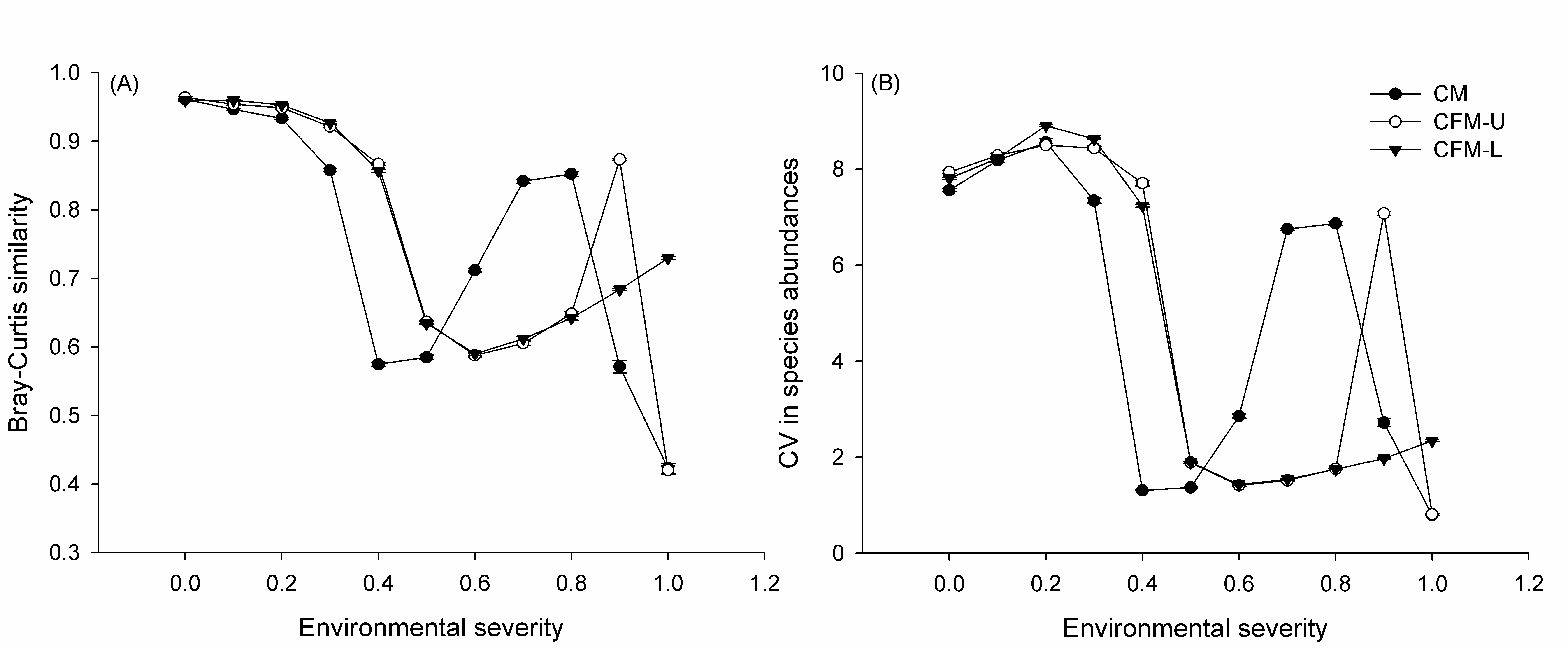

Supplement: Figure S3 — The Bray-Curtis similarity (A) and the coefficient of variation in species abundances (B) along the environmental gradient for communities with (CFM-U, and CFM-L) and without (CM) facilitation. The facilitative coefficient f = 0.3, and other parameter values are the same as in Fig. 1. Each data point represents the mean±SE. (TIF) [file pone.0078698.s003.tif]

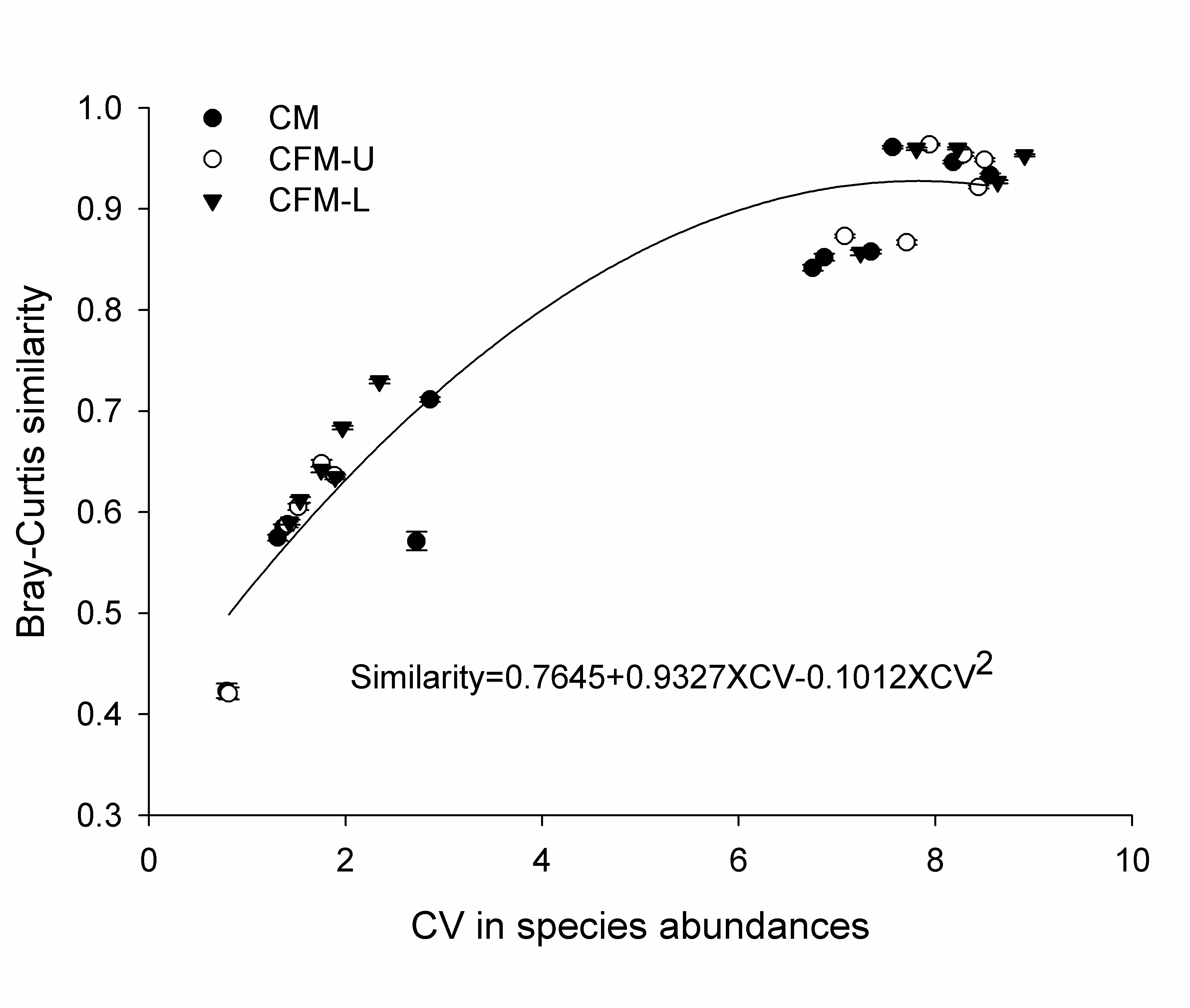

Supplement: Figure S4 — The relationship between the coefficient of variation in species abundances and the Bray-Curtis similarity index for communities with (CFM-U, and CFM-L) and without (CM) facilitation. The facilitative coefficient f = 0.3, and other parameter values are the same as in Fig. 1. Each data point represents the mean±SE. The R2 of the quadratic regression is 0.9230. (TIF) [file pone.0078698.s004.tif]

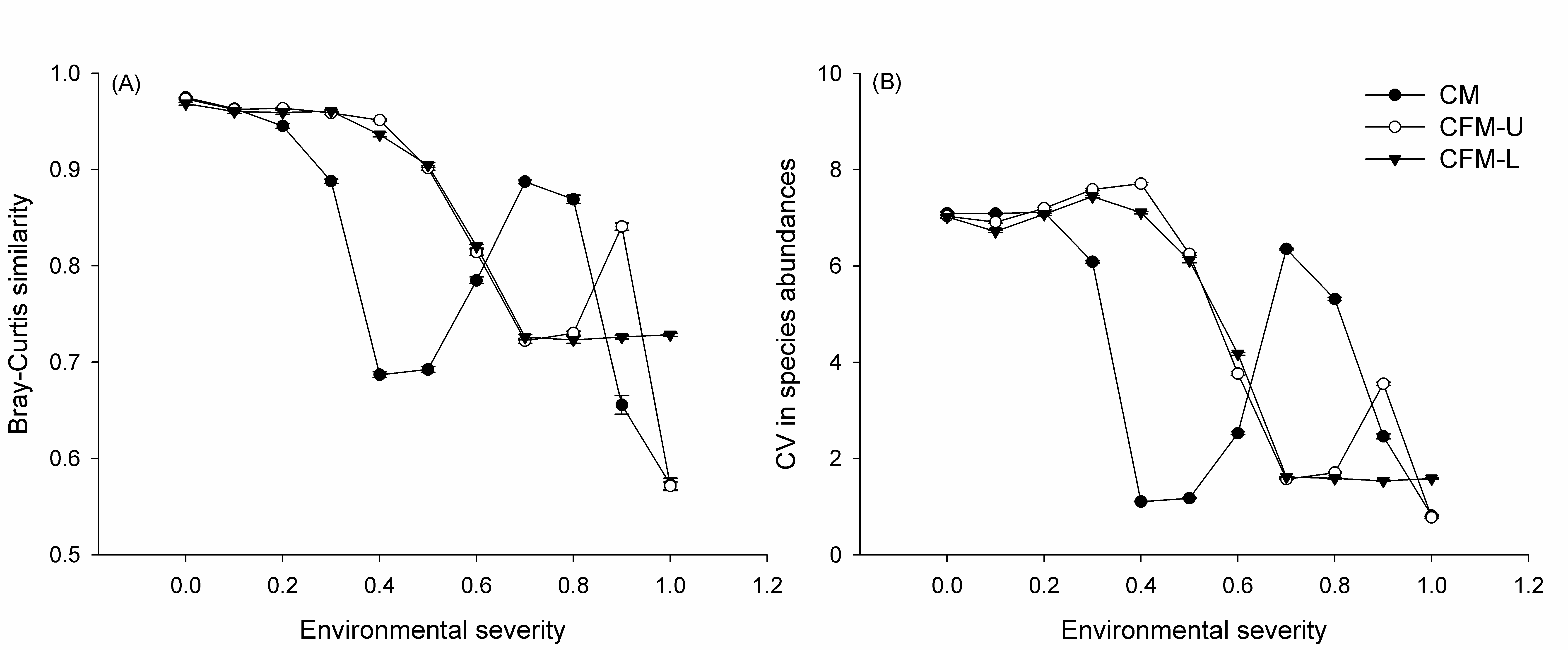

Supplement: Figure S5 — The Bray-Curtis similarity (A) and the coefficient of variation in species abundances (B) along the environmental gradient for communities with (CFM-U, and CFM-L) and without (CM) facilitation. The regional species pool R = 100, and other parameter values are the same as in Fig. 1. Each data point represents the mean±SE. (TIF) [file pone.0078698.s005.tif]

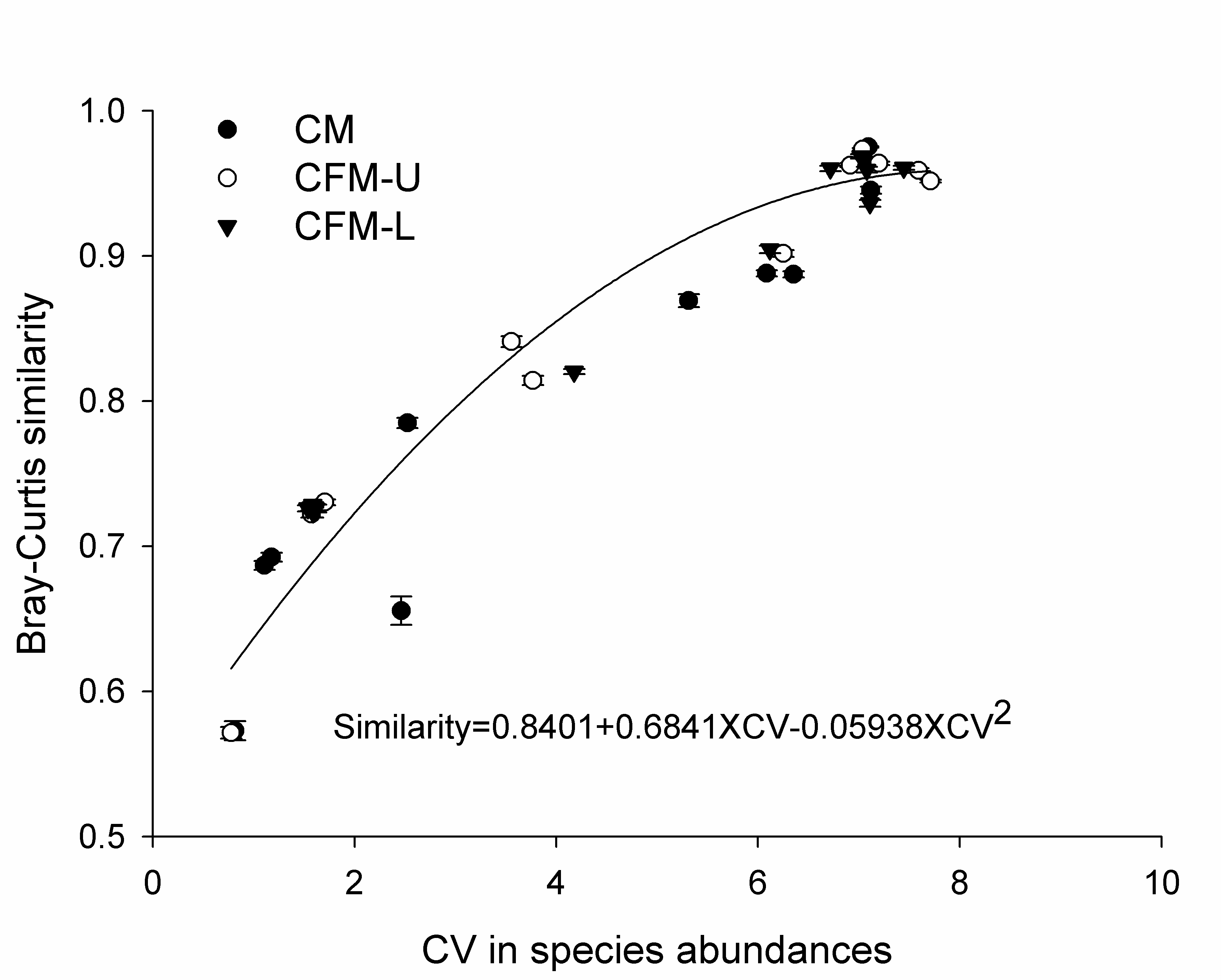

Supplement: Figure S6 — The relationship between the coefficient of variation in species abundances and the Bray-Curtis similarity index for communities with (CFM-U, and CFM-L) and without (CM) facilitation. The regional species pool R = 100, and other parameter values are the same as in Fig. 1. Each data point represents the mean±SE. The R2 of the quadratic regression is 0.9274. (TIF) [file pone.0078698.s006.tif]

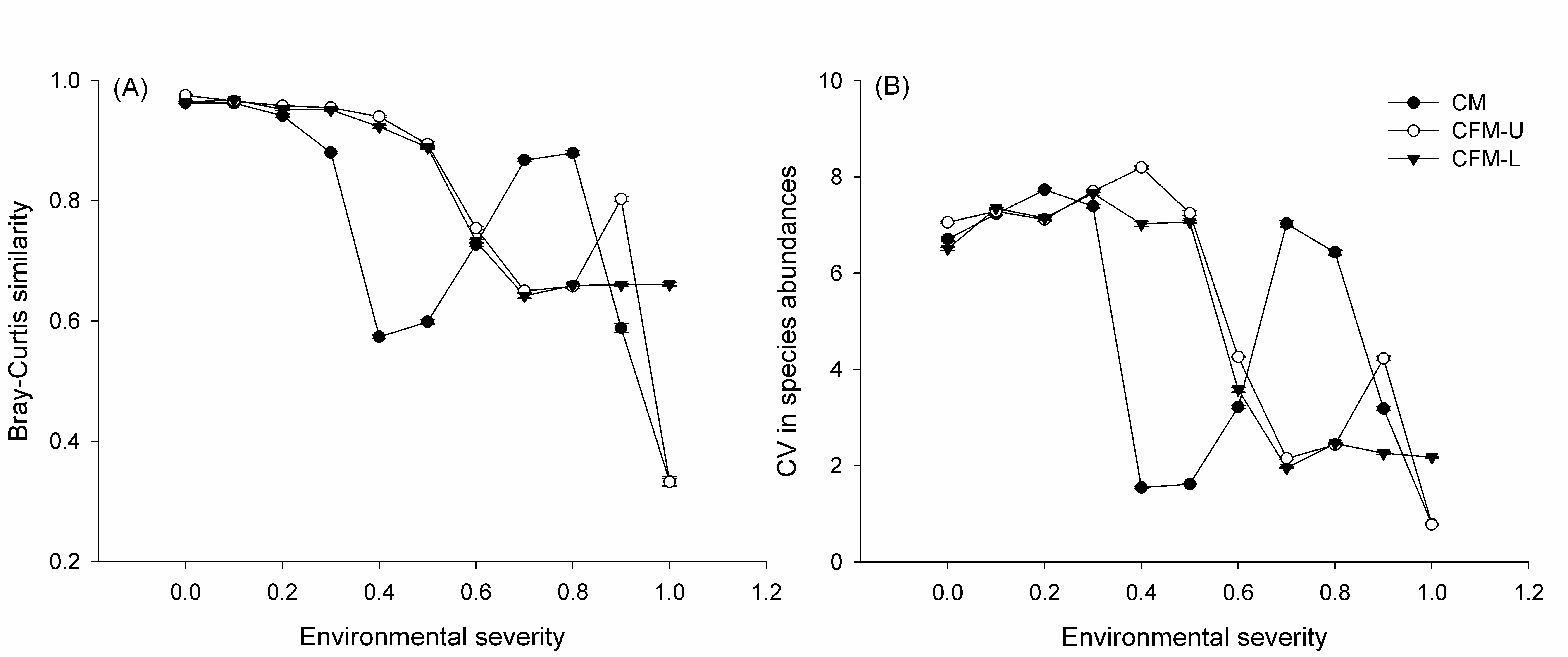

Supplement: Figure S7 — The Bray-Curtis similarity (A) and the coefficient of variation in species abundances (B) along the environmental gradient for communities with (CFM-U, and CFM-L) and without (CM) facilitation. The immigration rate I = 20, and other parameter values are the same as in Fig. 1. Each data point represents the mean±SE. (TIF) [file pone.0078698.s007.tif]

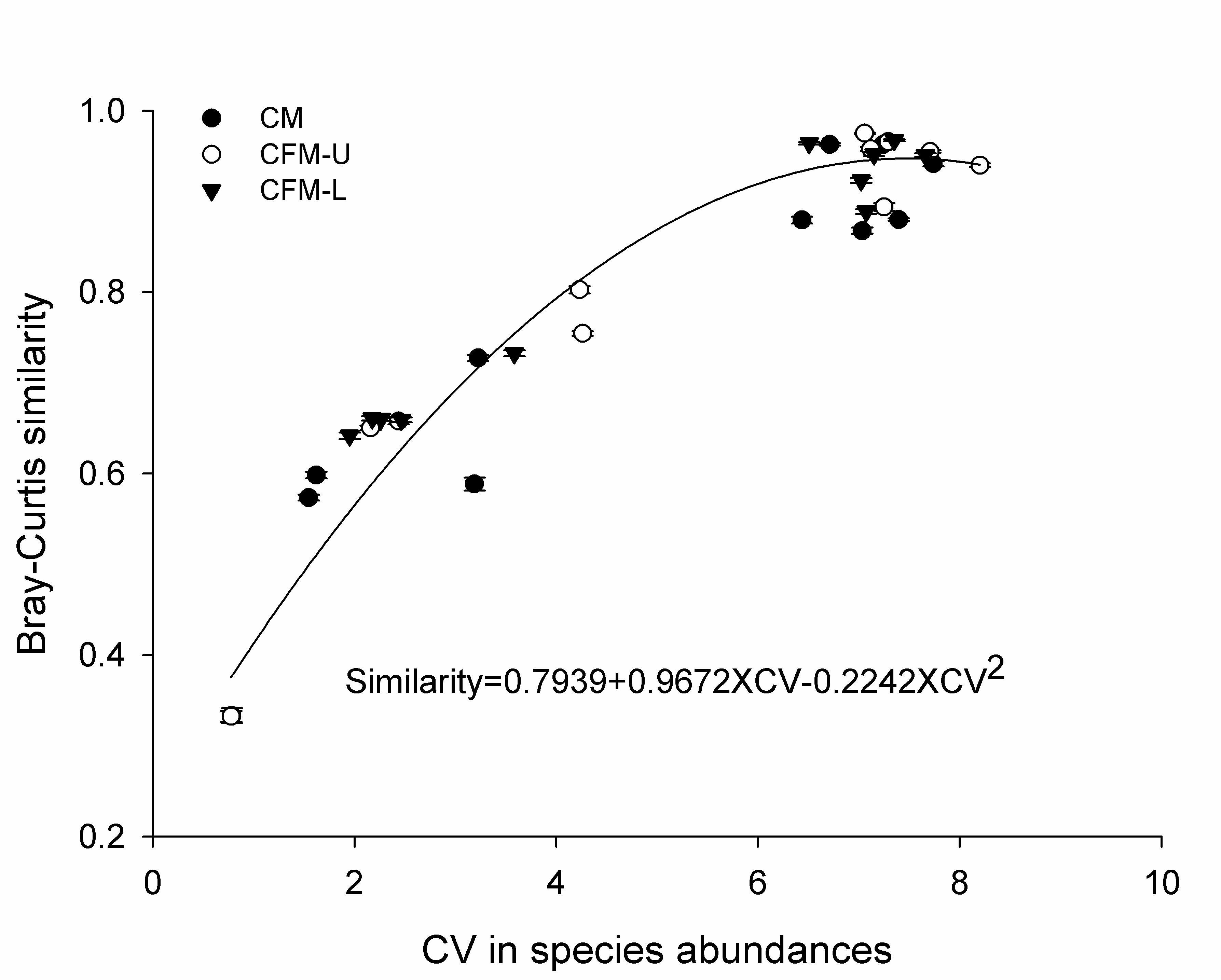

Supplement: Figure S8 — The relationship between the coefficient of variation in species abundances and the Bray-Curtis similarity index for communities with (CFM-U, and CFM-L) and without (CM) facilitation. The immigration rate I = 20, and other parameter values are the same as in Fig. 1. Each data point represents the mean±SE. The R2 of the quadratic regression is 0.9256. (TIF) [file pone.0078698.s008.tif]

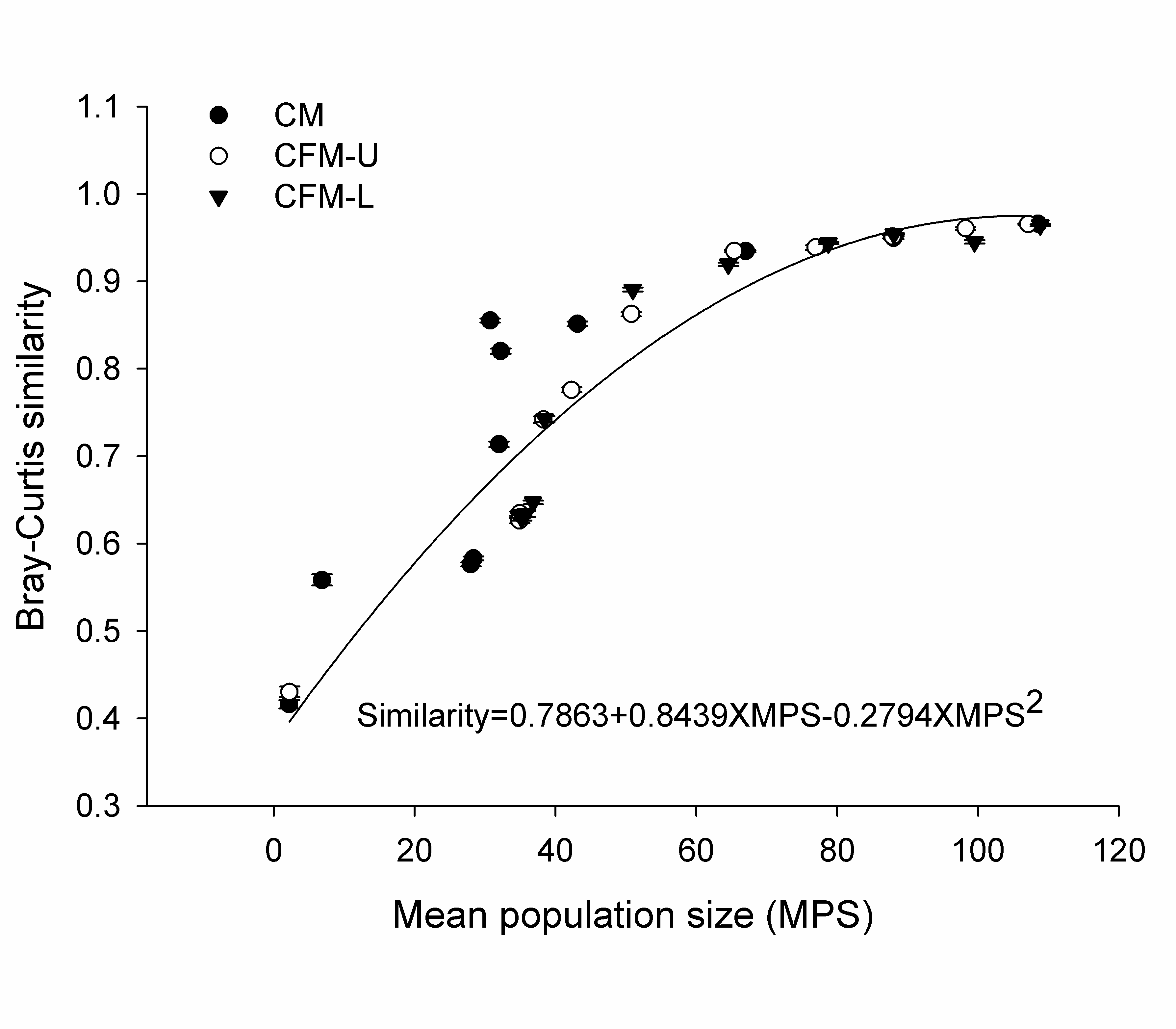

Supplement: Figure S9 — The relationship between the mean population size (MPS) and the Bray-Curtis similarity index for communities with (CFM-U, and CFM-L) and without (CM) facilitation. The parameter values are the same as in Fig. 1. Each data point represents the mean±SE. The R2 of the quadratic regression is 0.8585. (TIF) [file pone.0078698.s009.tif]
